# Bed bugs (*Cimex lectularius* L.) exhibit limited ability to develop heat resistance

**DOI:** 10.1371/journal.pone.0211677

**Published:** 2019-02-07

**Authors:** Aaron R. Ashbrook, Michael E. Scharf, Gary W. Bennett, Ameya D. Gondhalekar

**Affiliations:** Department of Entomology, Purdue University, West Lafayette, Indiana, United States of America; Universidade Federal do Rio de Janeiro, BRAZIL

## Abstract

The global population growth of the bed bug, *Cimex lectularius* (L.), is attributed to their cryptic behavior, diverse insecticide resistance mechanisms, and lack of public awareness. Bed bug control can be challenging and typically requires chemical and non-chemical treatments. One common non-chemical method for bed bug management is thermal remediation. However, in certain instances, bed bugs are known to survive heat treatments. Bed bugs may be present after a heat treatment due to (i) abiotic factors associated with the inability to achieve lethal temperatures in harborage areas for a sufficient time period, (ii) re-infestation from insects that escaped to cooler areas during a heat treatment or (iii) development of physiological resistance that allows them to survive heat exposure. Previous research has investigated the optimal temperature and exposure time required for either achieving complete mortality or sublethally affecting their growth and development. However, no research has examined bed bug populations for their ability to develop resistance to heat exposure and variation in thermo-tolerance between different bed bug strains. The goals of this study were: i) to determine if bed bugs could be selected for heat resistance under a laboratory selection regime, and ii) to determine if bed bug populations with various heat exposure histories, insecticide resistance profiles, and geographic origins have differential temperature tolerances using two heat exposure techniques (step-function and ramp-function). Selection experiments found an initial increase in bed bug survivorship; however, survivorship did not increase past the fourth generation. Sublethal exposure to heat significantly reduced bed bug feeding and, in some cases, inhibited development. The step-function exposure technique revealed non-significant variation in heat tolerance between populations and the ramp-function exposure technique provided similar results. Based on these study outcomes, the ability of bed bugs to develop heat resistance appears to be limited.

## Introduction

Of the ~100 species of blood feeding parasitic pests within the family Cimicidae, only the bed bug, *Cimex lectularius* (L.), and the tropical bed bug, *Cimex hemipterus* (F.), are associated with the recent global population resurgence [1, 2, 3]. Both, *C*. *lectularius* and *C*. *hemipterus* share hosts and their populations overlap in certain areas [1, 4, 5]. Yet, the ability of these organisms to tolerate environmental conditions influences their geographic distribution because they show differential temperature preference (*lectularius* 28–29°C, *hemipterus* 32–33°C) [1, 4, 6]. This allows for widespread distribution of *C*. *lectularius* in temperate regions, whereas *C*. *hemipterus* infestations are primarily in tropical/subtropical regions. However, both species have been recently found outside of the previously mentioned areas [1, 4, 7–9], likely because they are commonly found in stable indoor environments and are usually sheltered from the outdoor temperature extremes [10, 11].

Bed bugs are known to negatively influence humans as their bites can leave behind itchy red welts [1]. Elimination of bed bugs can be costly as it entails application of chemical insecticides and the use of non-chemical control techniques [12–14]. To avoid the challenges associated with locating all insects in an infestation, pesticide label restrictions on where a product can be applied within a residence and the potential for an insecticide resistant population to be present, whole residence heating is used for bed bug elimination [15–18]. Entire home heating is achieved by circulating heated air (55–65°C) indoors for six to eight hours with the ultimate goal of heating bed bug containing objects to >50°C [16]. Thermal remediation has many advantages. Not only can it eliminate all bed bug life stages within a residence, but it can also be used in areas or on objects where insecticides cannot be applied [15–18]. Additionally, setup of a heat treatment requires less preparation by occupants and it provides more immediate relief to them [16, 18]. However, there are also some drawbacks to the use of heat for bed bug disinfestation. For example, large scale heat treatments are time intensive, costly and do not provide any residual protection against bed bugs [16, 18]. Heat exposure may also damage temperature-sensitive items [16, 18]. Lastly, achieving the necessary lethal temperatures in thermally insulated areas such as cracks and crevices of walls or furniture where bed bugs prefer to reside is sometimes challenging.

If lethal temperatures are not achieved, bed bugs may detect and respond behaviorally to sublethally heated areas by fleeing to cooler areas such as wall voids, deep within furniture, or in neighboring unheated apartments [18–20]. Bed bugs stunned by sublethal heat exposure could fall into protected areas and recover afterwards [21]. In one case, it was observed that bed bugs escaped from a heat-treated apartment to an adjacent unheated unit to avoid heat exposure [20]. Loudon [22] reported that a single bed bug moved from the heated exterior to the cooler interior of a luggage case in an attempt to escape lethal heat exposure. Furthermore, when bed bugs are placed in an arena at room temperature (25°C), they can detect and orient towards a heated copper coil (28°C to 48°C) that is 10–30 mm away [23], which indicates they are good at responding to heated objects at short ranges. The abovementioned abiotic challenges in achieving lethal temperatures in harborage areas combined with the ability of bed bugs to behaviorally or physiologically respond to sublethal temperature exposure could theoretically select them for increased heat resistance.

There are several examples of arthropods adapting to temperature extremes. Heat exposing *Drosophila melanogaster* in the laboratory resulted in greater temperature resistance within a few generations of selection [24, 25]. Gray (2013) showed that plastic temperature tolerance traits can be selected within *Culex pipiens* if they are reared at different temperatures [26]. *Tetranychus cinnabarinus*, a greenhouse pest, was selected for resistance to abamectin and also showed some cross-resistance to heat exposure due to increased expression of heat shock proteins (HSP) [27, 28]. A springtail species, *Orchesella cincta*, was shown to significantly increase expression of the HSP70 family proteins after exposure to non-lethal high temperatures (heat hardening) prior to prolonged heat exposure [29]. Although bed bugs do not display heat hardening [30], repeated sublethal heat exposure could potentially select them for heat resistance, which would be problematic for the use of thermal remediation for their control.

Some of the previous temperature tolerance studies focusing on bed bugs have utilized two different exposure techniques. The first technique, “step-function”, is where the insects are exposed to a rapid increase in temperature [15, 30, 31]. The second technique is “ramp-function”, where the insects are exposed to a slow rate of rising temperatures [16, 30–32]. In another thermal biology study by Rukke et. al. [33, 34], the effects of rearing bed bugs at elevated temperatures (34 to 38°C) on survivorship, development and reproduction were reported. However, none of the previous studies have investigated different bed bug populations for variation in thermo-tolerance.

To address the knowledge gaps associated with the potential for bed bugs to develop heat resistance as well as the absence of data on variation in thermo-tolerance of different bed bug populations the goal of this research was two-fold. The first goal was to determine if a laboratory strain of bed bugs could be selected for heat resistance through sublethal heat exposure over multiple generations. The second goal was to utilize the step-function and ramp-function heat exposure techniques to evaluate the temperature tolerance of different bed bug populations.

## Materials and methods

### Insects

The insecticide-susceptible Harlan laboratory strain was used for heat selection experiments and as a reference population for thermo-tolerance comparisons. Information on the ten field populations used for heat tolerance screening are outlined in Table 1. Throughout this manuscript, the terms “strain” and “population” are used interchangeably. Field populations of bed bugs were collected from infested locations by pest management professionals (PMPs) and university researchers after obtaining verbal authorization from anonymous private property and business owners. No field studies were conducted for this research. All bed bug populations were maintained at 25 ±1°C, 50 ±10% RH and a 12:12 h (L:D) cycle in a temperature-controlled environmental chamber (Percival Scientific, Perry, IA). They were fed on defibrinated rabbit blood purchased from Hemostat Laboratories (Dixon, CA] using the membrane feeding method [35]. Heat selection experiments used large nymphs (4^th^–5^th^) that were starved for seven days prior to heat exposure (step-function technique). Similarly, adult bed bugs (1:1 male to female ratio) used for step-function and ramp-function experiments were fed seven days prior to their use. All field strains were laboratory-adapted and fed readily on defibrinated rabbit blood.

**Table 1:** Details of bed bug populations used in this study.

Strain name	Strain category	Collection State*	Collection Year	Year Tested
Harlan	Laboratory susceptible strain	New Jersey	1973	2015–2017
Hackensack	Pyrethroid treated before collection	New Jersey	2014	2017
KVS	Unknown	Florida	2006	2017
Bradenton	Unknown	Florida	2013	2017
Raleigh	Pyrethroid and heat treated	North Carolina	2013	2017
Lafayette	Pyrethroid, neonicotinoid, and heat treated	Indiana	2014	2017
McCall	Collected from a heat treated account	Florida	2016	2017
Richmond	Bifenthrin, deltamethrin, and chlorfenapyr resistant**	Virginia	2008	2017
Poultry House	Bifenthrin and chlorfenapyr resistant***	Tennessee	2013	2017
Knoxville	Bifenthrin, deltamethrin, and chlorfenapyr resistant***	Tennessee	2013	2017

* Information on the latitude of collection location strains is provided in S2 File

** Based on reference Ashbrook et al. [14] and Adelman et al. [36]

***Based on reference Ashbrook et al. [14]

### Heat resistance selection study

#### Determination of lethal time estimates for late instar nymphs of the Harlan strain

**Fig 1:**
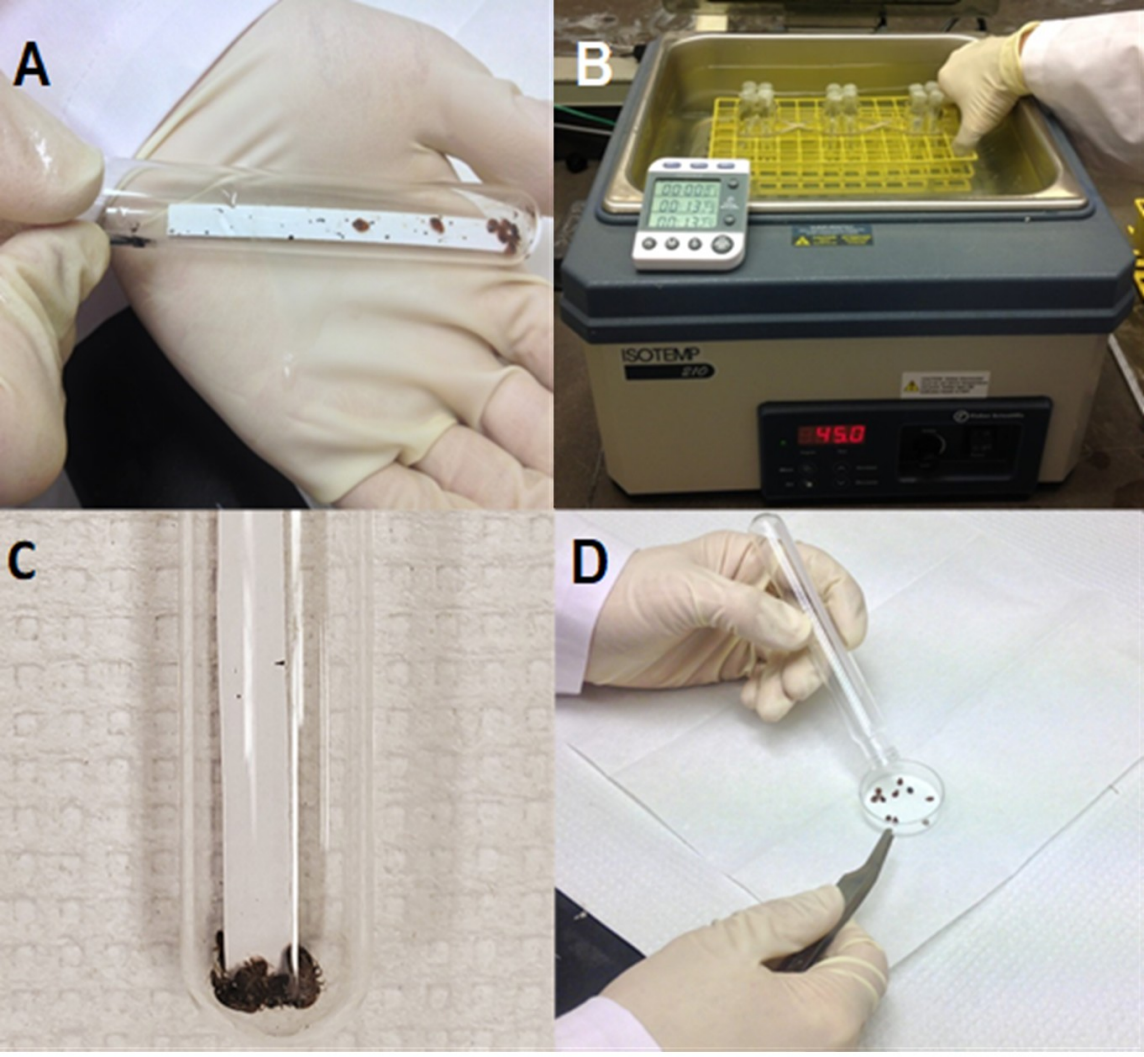
**A**. Bed bugs in a glass test tube with a strip of filter paper for harborage prior to heat exposure. **B**. An example of how the bed bugs were heat exposed in the water bath. **C**. After heat exposure in the water bath, the bed bugs were stunned and have fallen to the bottom of the test tube.** D**. Stunned bed bugs being placed in a Petri dish after heat exposure.

In order to select the Harlan strain for heat resistance, a LT_75_ (lethal time to kill 75% of the test population at 45°C) was determined for 4^th^–5^th^ instar nymphs by utilizing the step-function heat exposure method [15, 16, 29]. For the LT_75_ determination, ten Harlan strain nymphs were placed into a 15-mL glass test tube (Fisher Scientific, Pittsburg, PA) with a strip of notecard paper (Roaring Spring Paper Products, Roaring Spring, PA) for harborage (Fig 1A). Test tube openings were capped with Parafilm (Bemis NA, Neenah, WI). These tubes were then placed in a 12x6 plastic rack which was then placed in a water bath (Isotemp 210, Fisher Scientific, Dubuque, IA) heated to 45°C (Fig 1B). Rubber bands were used to secure the test tubes and prevent them from floating in the water bath. The exposure periods for nymphs in the 45°C water bath were 10, 12, 13, 14, 16, 17, 18, 20, 21, 22, 23, 24, 25 mins. After the exposure period had elapsed, test tubes were removed from the water bath and bed bugs were placed in a 35x10mm Petri dish (Fisher Scientific, Pittsburg, PA) with a Whatman No. 1 filter paper disc (GE Healthcare, Pittsburg, PA) (Fig 1D). Petri dishes were held in an environmental chamber with temperature, humidity, and light conditions identical to those used for rearing. Mortality was scored 24 h after exposure by prodding the insects with a toothpick. Insects were scored as dead if they could not move or right themselves after being prodded.

#### Selection regimen

The abovementioned step-function heat exposure method and the probit analysis-determined LT_75_ value (in mins) was used to select the Harlan 4^th^–5^th^ instar nymphs for heat resistance. An equal subset of nymphs not exposed to heat was maintained as a control colony. Each glass test tube that was used to confine bed bugs during heat exposure contained ten nymphs. Several test tubes were used for heat exposure experiments every generation depending upon the availability of nymphs. After heat exposure at the 45°C LT_75_ time, all nymphs from each individual test tube were transferred to a Petri dish with filter paper and mortality was scored after 24 h. Surviving nymphs from individual Petri dishes were then pooled in a single rearing container with mesh (Uline, Pleasant Prairie, WI), where they developed into adults and reproduced. Both control and heat-selected colony nymphs were fed one to two times weekly. The selection regime was continued from F0 to F7 generation (except F1) and initially began by selecting 300 nymphs (distributed in 30 test tubes) at F0 generation. As the selection process continued (F4 generation and beyond) less insects were used due to lower colony numbers. Therefore, depending on the availability of insects in each generation, between 50 and 300 nymphs were utilized for selection experiments.

### Assessment of blood-feeding and molting ability of heat exposed bed bugs

During the resistance selection procedure, heat-selected bed bugs were also qualitatively observed for sublethal effects such as the inability to feed to repletion and to successfully molt. Qualitative observations of the sublethal heat impacts on bed bugs led to conducting comparative experiments where the ability of heat exposed insects to feed was assessed. In order to quantitatively evaluate how heat affected blood feeding, 4^th^–5^th^ instar Harlan nymphs were exposed to LT_75_ time at 45°C and mortality was scored 24 h later. Survivors of heat exposure were then placed in jars and their ability to feed to repletion on defibrinated rabbit blood was observed on days five, eight, ten and fourteen after heat exposure. Identical numbers of control nymphs were placed in jars and also observed for their ability to feed to repletion at the same time points mentioned above. The number of insects utilized for each replicate was determined by the survival of the bed bugs in response to heat exposure at the LT_75_ time. Overall, six replicates were performed with an average of 40 bed bugs per replicate.

### Thermo-tolerance comparisons among bed bug strains

The procedures used for step-function thermo-tolerance comparison experiments were similar to those used for determining LT_75_ estimates for the Harlan nymphs. For each population, ten mixed sex adult insects (1:1 ratio) were placed into a 15-mL glass test tube with a strip of filter paper as harborage. Test tubes were sealed with Parafilm, placed in a 12x6 plastic holding rack and then transferred to a water bath heated to 45°C. Insects were exposed at 45°C for 10, 12, 13, 14, 16, 17, 18, 20, 21, 22, 23, 24, 25, 26, 27, 28, 29, and 30 mins to generate exposure time-mortality data. Three to four replicates (10 adults per replicate) were performed for each time point. Additional time points that provided 75–100% mortality were included in step-function heat exposure experiments to increase the precision of LT_99_ estimations [34]. Test tubes were removed from the water bath after the exposure period had elapsed and bed bugs were placed in a 35x10mm Petri dish with a Whatman No. 1 filter paper disc. Petri dishes were held in an environmental chamber with temperature, humidity, and light conditions identical to those used for rearing. Mortality was scored 24 h after exposure using the parameters described under determination of lethal time estimates for late instar nymphs. Control insects were held in test tubes at room temperature and then transferred to Petri dishes.

Procedures for the ramp-function heat exposure bioassay that utilizes a gradual or incremental increase in temperature were somewhat similar to those used for the step-function bioassays explained above. Briefly, 15-mL glass test tubes with 10 mixed sex adult bed bugs (1:1 ratio) per tube were placed in a 12x6 plastic holding rack that was transferred to a water bath at room temperature. The water bath was then turned on and the bed bugs were exposed to gradually rising temperatures at the rate of 0.57 °C/min until the water temperature reached 45°C. Once the water bath reached 45°C (~37-min heating time), insects were held in the water bath for a time that corresponded with the LT_99_ time for the Harlan strain. After the ramp-up heat exposure period was completed, insects were placed into Petri dishes with filter paper and mortality was scored 24 hours later using previously described criterion under determination of the lethal time estimates for late instar nymphs’ section. Three replicates of ten mixed sex bed bugs per replicate were performed for each population, including the Harlan strain, which was used as a positive control for all bioassay tests. Test tubes containing control insects were held at room temperature during the ramp-up heat exposure experiments.

### Data analysis

Time-mortality data for 4^th^–5^th^ late instar nymphs were utilized for PROC probit analysis in SAS 9.4 (SAS 2012, Cary, NC) to determine LT_75_ exposure time. The survivorship data for nymphs from the F0 to F7 generations were analyzed using ANOVA followed by all pairs Tukey’s test in SAS 9.4. Comparisons for feeding experiments were made in JMP 13.2 (SAS institute 2016, Cary, NC) using a repeated measures MANOVA with an interaction effect between nymphs and day. Nonparametric Wilcoxon tests were then conducted to determine if feeding response on any particular day was statistically different between heat-exposed and control insects. Exposure time-mortality data from the step-function experiments with adults was analyzed by PROC probit in SAS 9.4 to determine lethal time (LT_50_ and LT_99_) estimates and associated parameters for each population. The probit output values (intercept, slope, and covariance) were further used to statistically compare heat-tolerance profiles between different field populations as well as with the Harlan strain [37]. Mortality of field populations from the ramp-function heat experiments were analyzed by ANOVA using the PROC GLM function and means were separated using a Tukey’s test (*P<0*.*05*). Linear regression analysis was performed in JMP 13.2 to determine if there was any correlation between the LT_50_ or LT_99_ values and the latitude for the town/city where bed bug collections were made.

## Results

### Response of Harlan strain nymphs to heat selection

**Fig 2:**
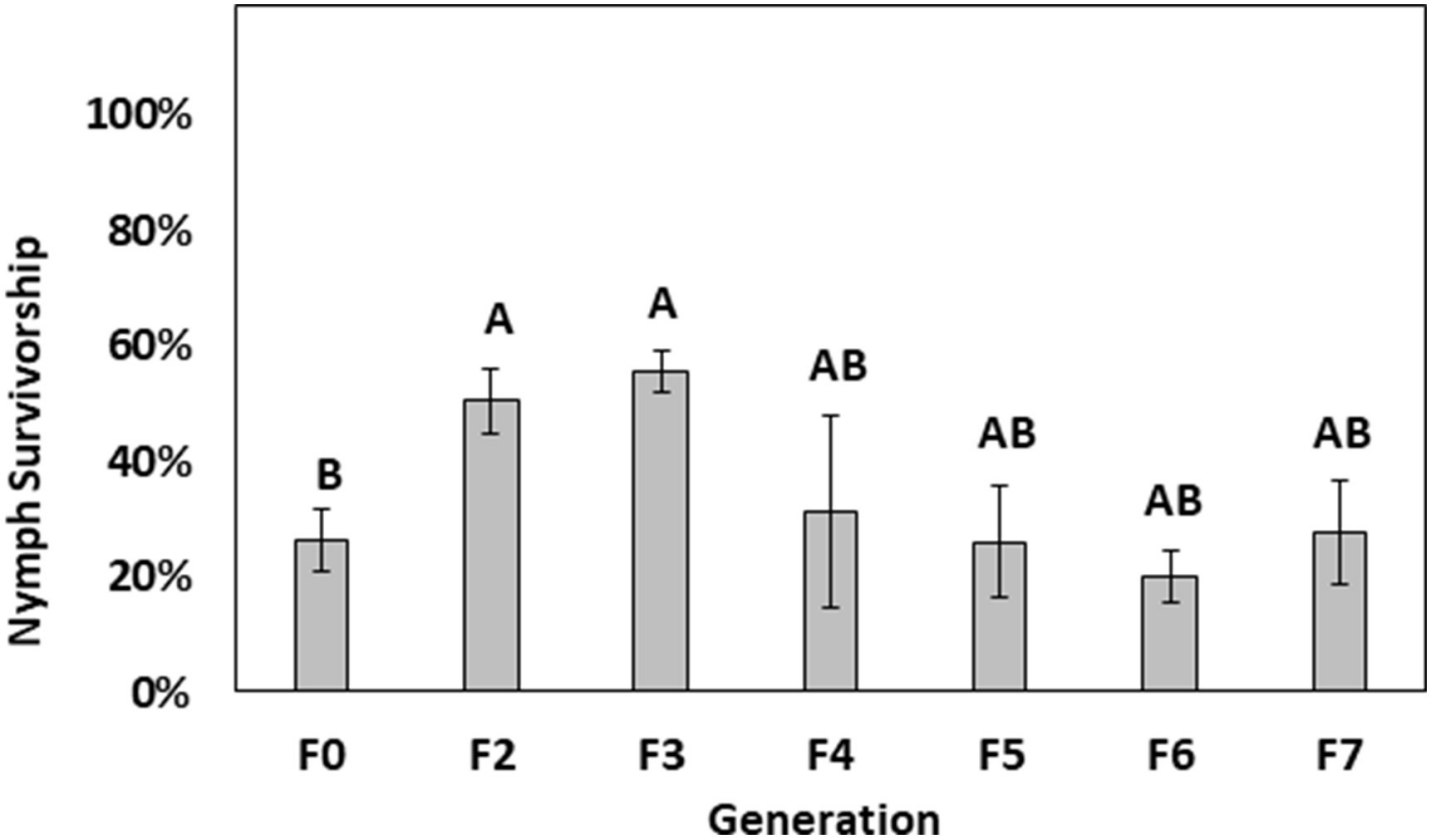
Bars depicting average survivorship of late-instar Harlan nymphs (4^th^–5^th^ instar) after each generation (F0 to F7) of selection or heat exposure at 45°C for 17.45 mins (LT_75_ time). Bars not connected by the same letter show statistically different survivorship rate (P<0.05; Tukey’s test).

Probit analysis conducted on time-mortality data for 4^th^–5^th^ instar Harlan nymphs indicated a time of 18.15 min at 45°C would kill 75% of test insects (LT_75_). However, for conducting selection experiments, the lower fiducial limit of the LT_75_ estimate (i.e., 17.45 mins) was used after performing empirical mortality validation tests, which showed that 17.45 min exposure caused ~75% mortality (Figure A in S1 File). The first round of selection (F0 generation) resulted in an average of 26.3% ± 5.5% of nymphs surviving (Fig 2). Survivorship significantly increased in the F2 and F3 generations to 50.5% ± 5.6%. and 55.5% ± 3.6%, respectively (ANOVA results: df = 35, 102, F = 1.63, P <0.001). However, survivorship in the F4 generation reduced to 31.4% ± 16% survivorship, which was statistically similar to the F0 generation. Exposure of F5 to F7 generation nymphs to the LT_75_ resulted in similar survivorship with an average of 26% ± 9.5%, 20% ± 4.4%, 27.8% ± 9.1% surviving the exposure. Although some of the F7 selected nymphs initially survived heat exposure, the attempt to establish the F8 generation was not successful because bed bugs died out completely likely due to the adverse effects such as reduced feeding and molting issues caused by the selection regime.

### Impacts of heat exposure on feeding and molting success

**Fig 3:**
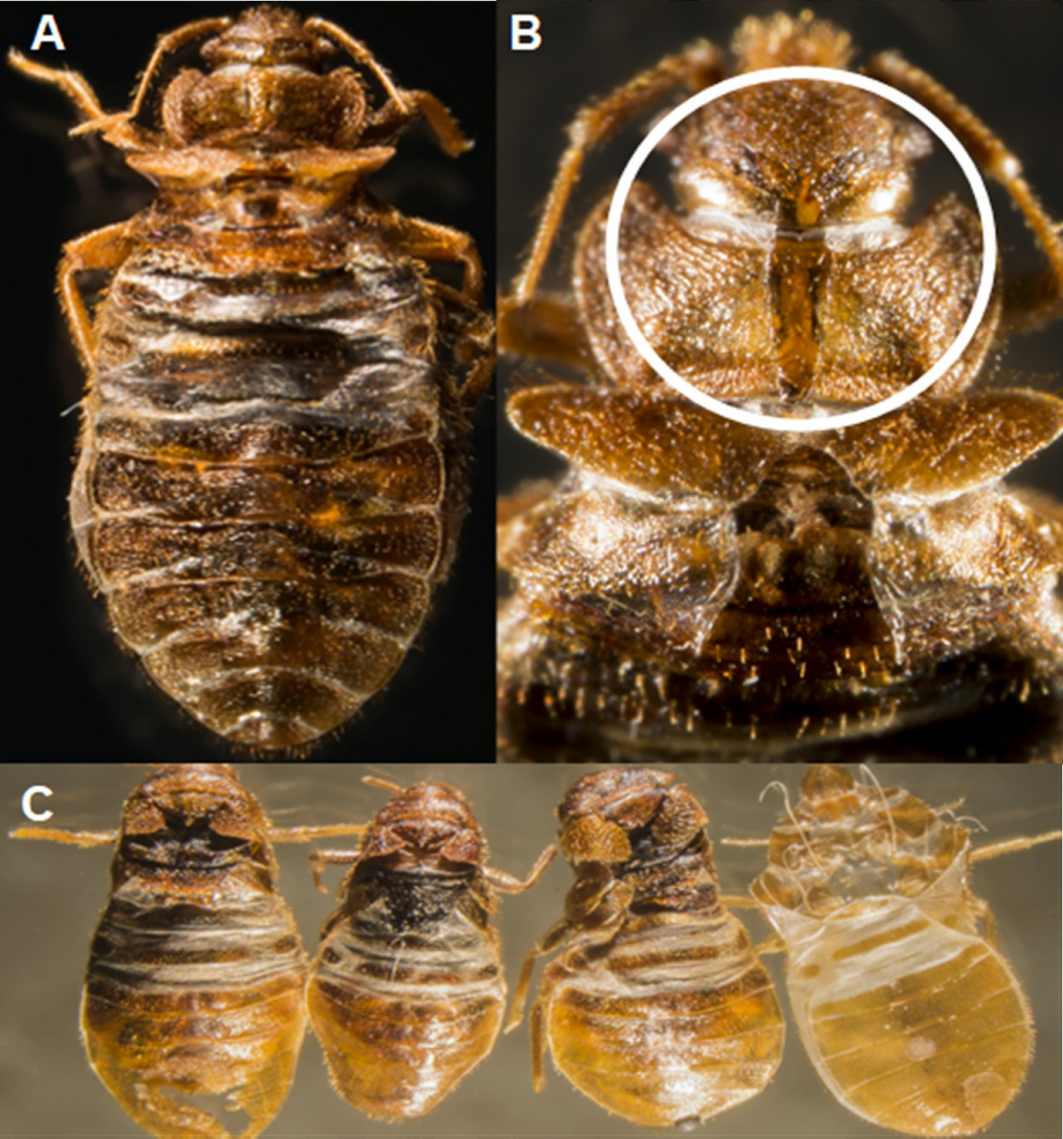
**A**. A large nymph that survived heat exposure, but was unable to complete the molting process. **B**. A magnified view of a heat exposed bed bug shown in the left image. This insect was attempting to molt, but failed to escape its exoskeleton. The epicranial suture is circled in white appears to have opened, but the bed bug failed to escape through it. **C**. Depicted in the image from left to right are three heat exposed nymphs that failed to successfully molt to next instar after heat exposure. On the right is an exuvia from a nymph that did successfully molt. Photo credit: John Obermeyer.

**Fig 4:**
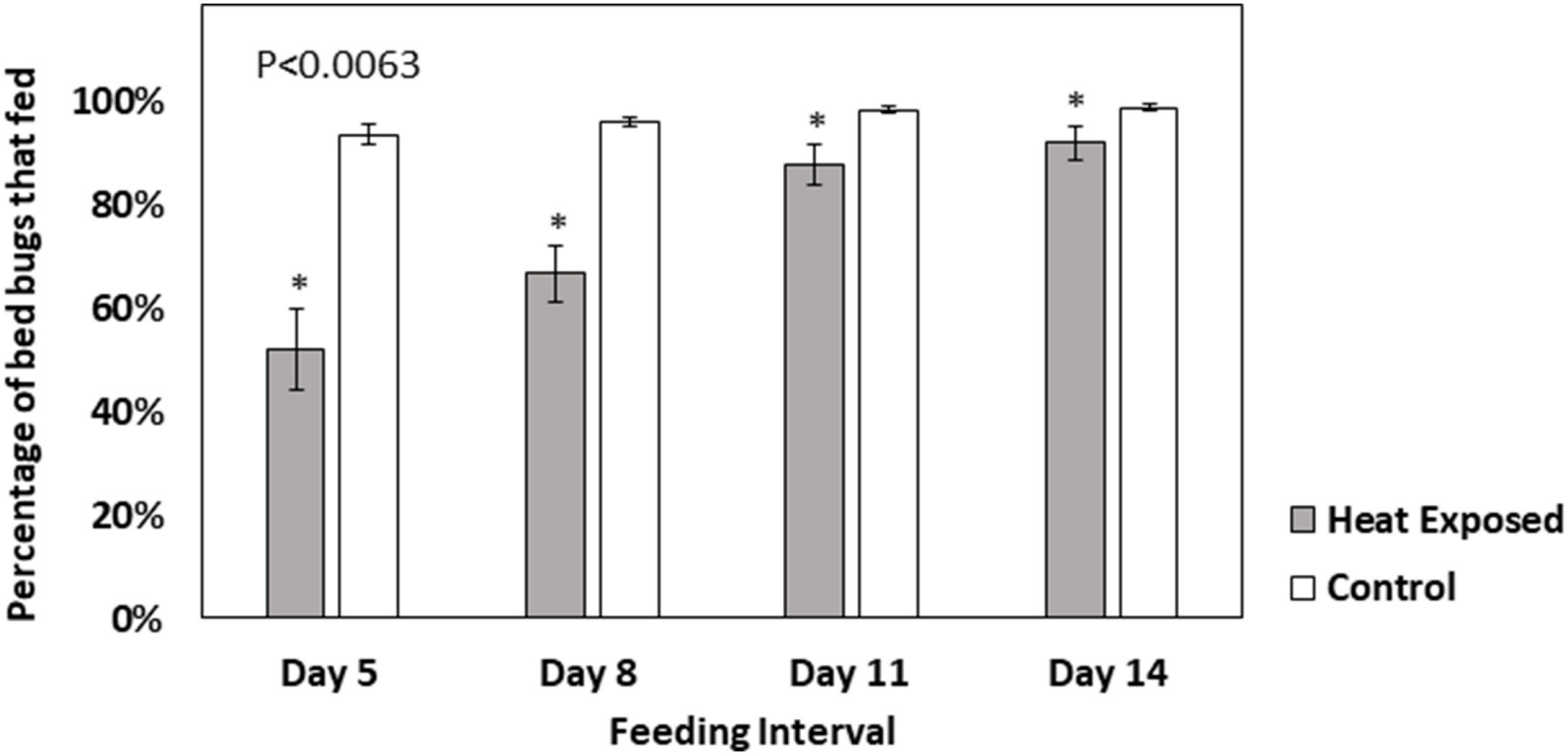
Bars representing percentage of Harlan nymphs from heat exposed (dark grey bars) and control (white bars) treatments that fed to repletion. Bed bugs that survived heat exposure at 45°C were offered blood meals at five, eight, eleven, and fourteen days after treatment (n = 40 per replicate). An equal number of control bed bugs that were not exposed to heat were offered a blood meal at the same time intervals. Statistically significant differences were found between the two treatment types and are denoted with an asterisks (*). Nonparametric Wilcoxon tests showed that at all feeding intervals feeding responses of heat-exposed and control nymphs were significantly different (P<0.05). Error bars indicate ± standard error (SE) values.

As mentioned above, some sublethal effects of heat exposure were observed in surviving insects. Initial qualitative observations suggested that fewer heat-exposed nymphs fed to repletion when offered a blood meal, but all control insects readily fed. Similarly, after feeding, some of the heat-exposed nymphs failed to escape from their exuvia and died during the molting process (Fig 3). The heat-exposed insects that died during molting showed dark pigmentation instead of the opaque and translucent appearance of normal teneral bed bugs. Molting defects were not observed in nymphs of the control strain. To verify the qualitative observations of reduced blood feeding by heat-exposed nymphs, a separate experiment was conducted where the feeding response of controls and nymphs that had survived heat exposure was quantitatively compared. Five, 8, 11, and 14 days after heat exposure, a significantly lower proportion of heat selected nymphs fed to repletion in comparison to the control strain nymphs (Fig 4, Repeated measures MANOVA results: df = 3, 8, F = 14.85, P <0.0012, Wilcoxon test results; day 5, Z = -2.80, P = 0.005; day 8, Z = -2.80, P = 0.005; day 11, Z = -2.74, P = 0.006 and day 14, Z = -2.77, P = 0.0055.).

### Heat tolerance comparison for different bed bug strains: Step-function method

The baseline LT_50_ and LT_99_ (and 95% fiducial limits) estimates for the Harlan susceptible strain adults at 45°C were 14.3 (13.7–14.8) and 23.21 (21.7–25.48) mins, respectively (Table 2). Empirical data showed that 100% mortality of the Harlan adults as well as 4^th^ – 5^th^ instar nymphs could be achieved with a 22-min exposure (Figures A and B in S1 File). Some differences were observed in the responses of different populations to heat exposure at the LT_50_ level, wherein the KVS strain showed significantly higher heat tolerance or resistance ratios in comparison to the Harlan, Raleigh, Hackensack, Richmond and Poultry House strains (P<0.05; Table 2 and Table A in S1 File). However, the LT_99_ values of the KVS strain were not significantly different from the Harlan and all field strains (P>0.05; Table 2 and Table B in S1 File).

**Table 2:** Lethal time (LT) estimates and probit output for bed bug populations exposed to 45°C.

Strain name	N	Slope (±SE)	LT_50_ (95% FL)*i*	LT_99_ (95% FL)*i*	Chi-square (df)
Harlan	540	11.02	14.3 (13.7–14.8)a	23.21 (21.7–25.5)a	11.6 (16)
Hackensack	540	11.39	16.5 (15.9–17.1)a	26.25 (24.7–28.4)a	12.7 (16)
KVS	560	13.46	19.7 (19.1–20.16)b	29.23 (27.8–31.2)a	11.05 (16)
Bradenton	550	11.89	17.6 (17.1–18.15)ab	27.63 (26.1–29.8)a	12.20 (16)
Raleigh	540	17.03	16.3 (15.9–16.6)a	22.30 (21.3–23.6)a	16.21 (16)
Lafayette	550	12.11	16.9 (16.4–17.5)ab	26.42 (24.9–28.4)a	14.81 (16)
McCall	550	12.42	17.1 (16.5–17.6)ab	26.25 (24.8–28.3)a	21.02 (16)
Richmond	560	9.73	15.1 (14.5–15.7)a	26.25 (24.5–28.7)a	8.84 (16)
Poultry House	550	8.28	14.5 (13.8–15.2)a	27.82 (25.7–30.8)a	16.6 (16)
Knoxville	540	11.57	17.4 (16.8–17.97)ab	28.31 (26.6–30.6)a	11.6 (16)

*i* Lethal time (LT_50_ and LT_99_) values with 95% fiducial limits (FL).

All values are expressed in mins. LT values within each column or category (i.e., LT_50_ or LT_99_) that are not connected by the same letter are significantly different as their confidence intervals do not overlap with the number “1” [37].

In spite of the lack of statistical support for differences in LT_99_ values for different strains, it was observed that strains with previous heat exposure histories Raleigh and McCall had lower LT_99_ estimates (22.3–26.3 mins) in comparison to some other populations such as Bradenton, Knoxville, KVS and Poultry House (LT_99_ of 27.6 to 29.2 mins). These populations with the highest LT_99_ values also tended to have the highest predicted LT_50_ values, except the Poultry House strain, which had an LT_50_ value close to that of the Harlan strain. No correlation was observed between the latitude of collection location and the LT_50_ or LT_99_ estimates for different field strains (LT_50_: R^2^ = 0.19, P > 0.21, LT_99_: R^2^ = 0.23, P > 0.16). Similarly, LT_50_ and LT_99_ estimates of the strains with documented history of insecticide resistance (Richmond, Knoxville and Poultry house) were not significantly different than that of the susceptible Harlan strain (P>0.05; Table 2, Tables A and B in S1 File). Lastly, no control mortality occurred in any of the bioassay experiments.

### Heat tolerance comparisons for different bed bug strains: Ramp-function method

No variability was found in temperature tolerance of bed bug populations in the ramp-function heat exposure bioassays conducted at temperatures between 25 to 45°C (data not shown). Complete (100%) mortality was achieved for all strains (ANOVA results, df = 9, 20, P > 0.99) including the Harlan population. No mortality was observed in untreated controls.

## Discussion

### Factors affecting heat resistance development in bed bugs

When inside a human dwelling, bed bugs face a variety of challenges, such as starvation, desiccation, damage by traumatic insemination and local extinction through the implementation of pest management strategies. In comparison to other control strategies such as the use of insecticides, how bed bug populations respond to thermal challenges has been less studied. Late instar (4^th^–5^th^) nymphs were utilized to determine if a *C*. *lectularius* laboratory population could develop heat resistance. This life stage was chosen because the 4^th^–5^th^ nymphs are close in size to adults, but are still sexually immature. Therefore, these individuals were capable of reproduction only if they survived heat selection and successfully molted to the adult life stage. Additionally, no significant differences in temperature tolerance were observed between late instar nymphs and adults (Figures A and B in S1 File).

The Harlan population was selected for heat resistance by exposing them to a pre-determined LT_75_ over the F0 to F7 generations (Fig 2). During the selection regime, increased survivorship was initially seen for the F2 and F3 generations. However, when F4 nymphs were heat selected, their survivorship decreased relative to previous generations. In subsequent generations (F5 to F7), survivorship declined further. Although some insects initially survived the heat selection in the F7 generation, none survived long enough to establish the F8 generation and eventually selection could not proceed further. Previous heat selection experiments with other insect species have used a variety of techniques to determine if selection for heat resistance is possible. Laboratory experiments that used ramp-function heat to select *D*. *melanogaster* found a significant increase survivorship up to the F4 generation; however, survivorship was not reported after this generation [25]. When two *D*. *melanogaster* populations were reared at different temperatures for 4 years, the population reared at higher temperature was better at tolerating step-function heat exposure [24]. However, rearing bed bugs at temperatures greater than 30°C in order to select them for temperature tolerance would likely not select them for heat resistance since research has shown that rearing bed bugs at these temperatures causes mortality, sterility, and developmental issues [33, 34, 38].

The initial increase in survivorship followed by a decline in survivorship indicates that bed bugs may have a limited ability to develop greater temperature resistance in a laboratory setting. This could be due to many factors. One of the factors affecting the ability of bed bugs to develop heat resistance, could be the lack of genetic diversity in a laboratory colony (Harlan strain). Adapting insects to laboratory conditions can reduce the genetic diversity of a population compared to wild type populations, which has previously occurred with the sandfly, *Lutzomyia longipalpis* [39]. Genetic diversity of laboratory colonies such as the Harlan strain can also be reduced when in culture for a long duration. Kim et al. [40] found that the Western corn rootworm had decreased genetic diversity when in culture for ~190 generations. Similarly, older laboratory colonies of *D*. *melanogaster* experience reduced genetic diversity in comparison to recently established colonies [41]. Additionally, genetic studies on bed bugs have found that field populations have low genetic diversity within populations [42] and the Harlan population is likely no different. Given the low genetic diversity within different bed bug populations in general, the findings of the laboratory selection study likely also hold true for field populations, i.e., the ability of bed bugs to develop stable and significant levels of heat resistance in a field setting could be very limited.

In addition to the low genetic diversity within bed bug populations, the sublethal effects of heat exposure observed in this study, which were consistent with other studies [15, 33, 34, 38], may further constrain the ability of bed bugs to develop heat resistance. Similar to previous research [15] when bed bugs were exposed to sublethal heat they were initially stunned (Fig 1C) and could not walk, but some recovered and were capable of movement after 24 h. Bed bugs require blood meals in order to molt and reproduce successfully [1]. However, heat-exposed bed bugs showed a significantly reduced feeding preference relative to control nymphs for up to 14 days post exposure (Fig 4). Similarly, in another study, reduction in bed bug feeding was observed after exposure to sublethal levels of steam [43]. Bed bugs that did not feed after heat exposure could have been avoiding further stress associated with consuming a hot blood meal. Blood feeding has been shown to increase the body temperature and elicit HSP expression in mosquitoes [44]. It has also been reported that bed bugs that feed on overheated blood (39°C) will die, likely due to heat stress [18]. Although not investigated in this study, the heat selection regime may also have impacted bed bug reproduction by eliminating *Wolbachia* symbionts [38]. It has been previously shown that rearing bed bugs at 36°C can significantly reduce *Wolbachia* cell counts from their mycetomes, which consequently reduces egg viability for up to 10 weeks after exposure [38]. However, if bed bugs are briefly exposed to steam, their reproduction is not impacted [43]. This indicates that bed bugs must be heat-exposed for a longer duration to eliminate their *Wolbachia* symbionts. In the future, quantitative PCR experiments could be conducted to determine the heat exposure duration required to eliminate the microbial symbionts of bed bugs using the step-function or ramp-function methods.

In some instances, we found that nymphs that survived the step-function heat exposure failed to escape from their exuvia during molting (Fig 3). Experiments with the flesh fly, *Sarcophaga crassipalpis*, found that some adults were unable to successfully eclose from the puparium after sublethal heat exposure [45]. Similar to findings mentioned above, Rukke et al. [34] reported that *C*. *lectularius* nymphs reared at temperatures between 34 –; 38°C for two to three weeks failed to molt properly. Studies with other arthropods have shown that physiological adjustments required for overcoming heat stress also have deleterious effects on reproduction and development [45–48].

The deleterious effects of heat exposure on bed bugs, such as reduced blood feeding and molting abnormalities, likely became an important factor regarding survivorship and developmental ability of the heat-selected strain beyond the F4 generation (Fig 2). Eventually, the heat-selected colony died out completely after the F7 heat exposure experiment. If the heat-associated sublethal effects of this study are extrapolated to the field, the heat-exposed bed bugs that survive may be less successful in passing their genes to the next generation, which would further reduce the probability of heat resistance evolution.

### Minimal variation in thermo-tolerance of bed bug strains

The final goal of this study, was to test the ability of field strains to tolerate heat using both the step-function and ramp-function heat exposure techniques (Tables 1 and 2, Tables A and B in S1 File). Another objective of these experiments was to determine the influences that geographic origin, insecticide resistance status and previous heat exposure history have on temperature tolerance of bed bug field strains. Adult bed bugs (1:1 ratio of males and females) were used for the thermo-tolerance bioassays because they are one of the most temperature tolerant among the mobile life stages [49]. The temperature tolerance of early instar nymphs was not determined. However, *C*. *hempiterus* first instar nymphs have lower temperature tolerance in comparison to adult *C*. *hempiterus* [49] and *C*. *lectularius* may be similar in this regard. Additionally, bed bugs starved for 7 d prior to heat exposure that were used in this study were likely close to an optimal thermo-tolerant state [11]. Previous research has shown that bed bugs that were fed 1 d and 21 d prior to heat exposure are less thermo-tolerant than insects fed 9 d fed prior to heat exposure [11]. Devries et al. [50] suggest that there is a metabolic state around this optimal feeding status that maximizes bed bug thermo-tolerance, but what causes this relationship between thermo-tolerance and metabolism is unclear.

Using the step function technique, some variability was observed in LT_50_ times (Table 2 and Table A in S1 File), however, none of the LT_99_ estimates were significantly different (Table 2 and Table B in S1 File). No clear patterns emerged with respect to the LT estimates and previous history of heat exposure, geographic origin or insecticide resistance status. In comparison to other strains, the bed bug populations that had a history of heat exposure did not show significantly higher LT_99_ values (e.g., Raleigh, NC, LT_99_ 22.3 min, McCall, FL, LT_99_ 26.3 min). This could have been due to the variety of demonstrated impacts of heat exposure found in this study as well the fitness costs documented in other insect species [15, 33, 34, 38, 43–48]. Secondly, the geographic origin (latitude of collection location) of a bed bug population also did not influence their temperature tolerance, likely since indoor environments are relatively stable and based on the preference of the tenant. Bed bugs thus are probably not exposed to sufficiently variable temperatures over many generations to change their thermo-tolerance. In Japan, a study with 30 different *Drosophila* species found that the temperature tolerance did not vary by the geographic latitude of a population, but rather the habitat type (e.g., tree canopy versus open field conditions) [51]. Lastly, pyrethroid resistant strains (e.g., Knoxville, Lafayette and Richmond) [14, 36], did not show significantly different thermo-tolerance based on the LT_50_ and LT_99_ values in comparison to the Harlan strain (Table 2, Tables A and B in S1 File) indicating lack of correlation between insecticide resistance status and heat tolerance. However, because of the unknown insecticide resistance status of the KVS strain that shows significant thermo-tolerance at the LT_50_ level, we could not confirm if heat tolerance of this strain is associated with pesticide resistance. Previously, abamectin (an avermectin class insecticide) resistant mites were also shown to have cross-resistance to heat [27, 28] but since this insecticide is not used for bed bug control, it is likely that they would not develop cross resistance to heat in this way.

The absence of any significant differences in the thermo-tolerance among bed bug populations were further verified using the ramp-function exposure technique. With the ramp-function technique, the temperature is gradually increased, which is similar to how heat is deployed in the field [16, 31]. This method also allows bugs more time to physiologically respond to thermal stress. However, complete mortality was achieved in all bed bug populations that were tested using the ramp-function technique. It is possible that during the process of establishing colonies of wild type bed bugs in a laboratory setting, the insects may have gone through a significant bottleneck effect that could have further reduced or eliminated any substantial differences in thermo-tolerance that were originally present. Additionally, how arthropods express heat shock proteins, other stress-induced genes, and metabolites such as sugars and amino acids in a field setting in response to thermal challenges is not well understood. Instead of increasing expression of HSPs and stress-induced genes to survive heat exposure, a more optimal response could be to flee to cooler areas to avoid heat stress, and this appears to be the case when bed bugs are exposed to heat [52]. Bed bugs express heat shock proteins when heat exposed and it has been shown that they have 13 *HSP* genes [44, 53]. However, *HSP* gene expression profiles for the bed bug populations used in this study in response to heat exposure are yet to be determined.

With respect to the role of metabolites in thermal tolerance, *Belgica antartica* is known to increase internal concentrations of trehalose to become more tolerant to both heat and cold [54]. Arthropods can also increase the proportion of saturated lipids and cuticular hydrocarbons (e.g., *n*-alkanes) in their cell membrane and cuticles, respectively, to help reduce water loss and aid in temperature tolerance [55, 56]. In response to rising environmental temperatures, *Orchesella cincta* can increase the proportion of saturated lipids in their cellular membranes [57]. Similarly, when *Pogonomyrex barbatus* were exposed to higher temperatures and lower humidity for 20 days, they increased the proportion of saturated cuticular hydrocarbons in their exoskeleton [58]. Bed bugs are similar to desert-adapted arthropods in their ability to withstand desiccation [59] and have also shown the ability to evolve modified cuticles to resist insecticides [60]. However, the roles of metabolites (trehalose) and changes in cuticular hydrocarbon profiles in bed bug heat tolerance are not known and should be further investigated.

It is possible that small differences in LT_50_ and LT_99_ durations of different populations (Table 2), although mostly non-significant, could allow some populations such as KVS, Poultry house, and Bradenton to escape insufficiently heated areas in the field more effectively than other bed bug populations. Research indicates that if bed bugs are exposed to sublethal temperatures or if the heat in an area is uneven, they would move to an area with more suitable temperatures [18, 19]. Currently, the bed bug strains tested in this study are being examined for differences in their heat repellency behavior by exposing them to rising environmental temperatures in harborages that are gradually heated (ramp-function method.

### Implications for bed bug control

The range of sublethal impacts caused by heat exposure as well as the upper physiological limits of *C*. *lectularius* heat tolerance has implications for using lethal heat as a control measure for bed bug elimination. First, if bed bugs remain after a heat treatment or are present in a follow-up inspection, the chances that these insects have developed any substantial heat resistance are low. The initial increase followed by a decrease in bed bug survivorship during heat selection experiments in addition to the plethora of sublethal heat impacts, suggest that individuals that are more heat resistant are quickly selected against (negative selection in a few generations). An alternative explanation for insects remaining after a heat treatment is that they were exposed to sublethal temperatures, escaped from high temperature zones, or were re-introduced to the domicile [18]. If the resident complains of being bitten by bed bugs shortly after a heat treatment, the latter explanation is likely; given that heat-exposed bed bugs will feed at reduced rates for up to two weeks.

In order to ensure that all insects are eliminated within an infestation, temperatures ≥ 50°C as well as a sufficient exposure period are required [16], especially if bed bugs are suspected to be harboring deep within objects. Monitoring temperatures throughout heated areas in order to identify heat sinks and/or insulated areas is critical for complete bed bug elimination [18]. Since bed bugs have been shown to travel long distances within an infestation [61] and can detect heated objects [23], they will likely flee to cooler spots or adjacent housing units if sublethal temperatures are used during thermal remediation [18, 19]. Therefore, interception measures should be utilized to trap bed bugs within areas that are heated to 50°C or higher. Placing traps, sealing wall cracks or electrical outlets, and applying silicate dusts or insecticides to create a barrier would prevent bed bugs from escaping. Additionally, if there are areas that are not reaching temperatures ≥50°C, then insecticides can later be applied as spot treatments to those areas and other control strategies can be deployed [18]. This is a well-known practice that is already utilized by some pest management companies. It is important to note that heat is one of many tools available for bed bug elimination and should be deployed with other IPM strategies and insecticides to maximize control.

## Supporting information

S1 File Supporting Figures and Tables. (PDF)

S2 File Raw data from all experiments. (XLSX)
