# Supplementary material for: Bed bugs (Cimex lectularius L.) exhibit limited ability to develop heat resistance
Source: PLoS One. 2019 Feb 7;14(2):e0211677. doi: 10.1371/journal.pone.0211677 (PMC6366730; doi:10.1371/journal.pone.0211677)
Supplement: S1 File — (PDF) [file pone.0211677.s001.pdf]

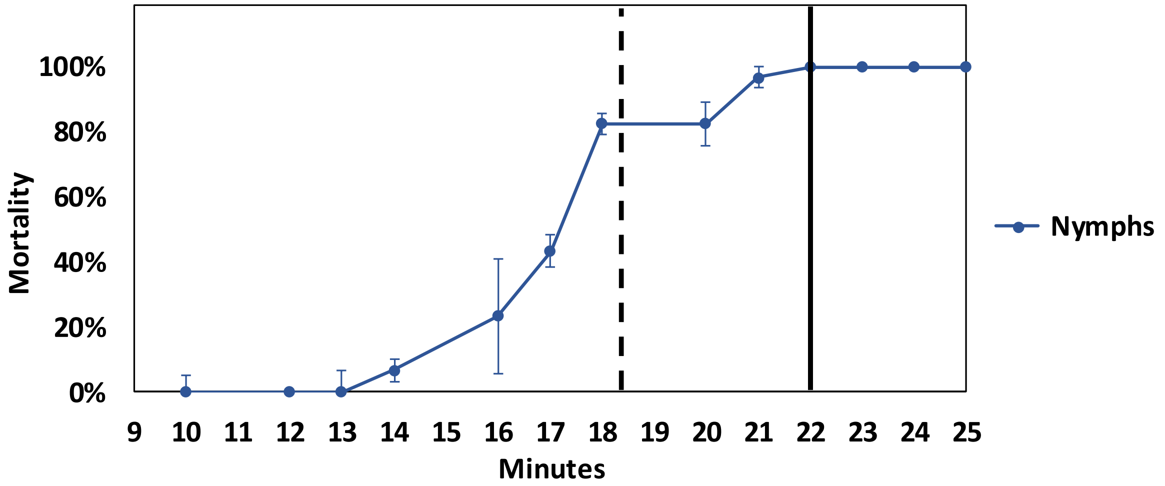

Figure A. Harlan nymph time-mortality curve. Time-mortality data for step-function bioassays with Harlan nymphs. All insects were exposed to 45 °C and the exposure times at which they were tested are expressed in minutes. The first dashed vertical line denotes the LT_{75} exposure time for nymphs, whereas the second black line represents the time at which 100% nymph mortality was achieved.

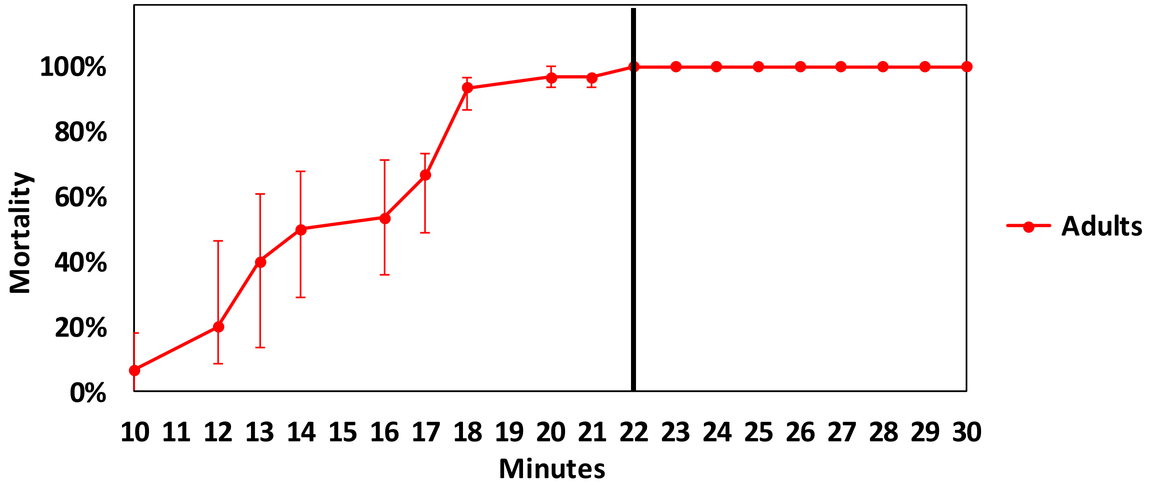

Figure B. Harlan adult time-mortality curve Time-mortality data for step-function bioassays with Harlan adults. All insects were exposed to 45°C and the exposure times at which they were tested are expressed in minutes. The solid vertical line indicates the time at which 100% mortality was first observed for adult bed bugs (22 minutes). Results from a statistical analysis test [37] indicated that the life stages (nymphs and adults) were statistically similar to each other. Comparative temperature tolerance ratios between nymphs and adults of the Harlan strain are close to or overlapped with the number one {TR_{50}= 0.84 (0.72–1.00), TR_{99}= 1.09 (0.77–1.53)}, indicating that their thermo-tolerance levels were similar. Error bars indicate (±) standard error values.

Table A. Comparison of field strain probit output at the LT_{50} level. Resistance ratios (RRs) at LT_{50} and corresponding confidence intervals (in parenthesis) for bed bug strains exposed to 45°C. Significance of RRs was determined using the statistical test described by Robertson et al. [37]. Strains in each column were individually compared to the strains in each row. Within each column RRs that are not connected by the same letter are statistically different. RRs are significantly different if their confidence intervals do not overlap with the number "1". No control mortality occurred during these experiments.

**Strain name**	Harlan	Raleigh	McCall	Hackensack	Richmond	Lafayette	Bradenton	Poultry House	Knoxville
Harlan	-	-	-	-	-	-	-	-	-
Raleigh	1.14 (1.0–1.33)a	-	-	-	-	-	-	-	-
McCall	1.19 (1.0–1.42)a	1.05 (0.91–1.2)a	-	-	-	-	-	-	-
Hackensack	1.16 (1.0–1.38)a	1.01 (0.88–1.17)a	0.97 (0.82–1.14)a	-	-	-	-	-	-
Richmond	1.06 (0.89–1.27)a	0.93 (0.80–1.08)a	0.89 (0.75–1.05)a	0.92 (0.77–1.08)a	-	-	-	-	-
Lafayette	1.19 (1.0–1.41)a	1.04 (0.91–1.2)a	0.99 (0.85–1.17)a	1.03 (0.87–1.21)a	1.12 (1.0–1.32)a	-	-	-	-
Bradenton	1.23 (1.0–1.46)a	1.08 (0.94–1.68)a	1.03 (0.88–1.21)a	1.07 (0.91–1.25)a	1.0 (0.72–1.40)a	1.04 (0.89–1.21)a	-	-	-
Poultry House	1.17 (1.0–1.37)a	0.9 (0.77–1.0)a	0.85 (0.72–1.0)a	0.88 (0.74–1.0)a	0.96 (0.8–1.15)a	0.86 (0.72–1.0)a	0.83 (0.7–1.0)a	-	-
Knoxville	1.22 (0.86–1.72)a	1.07 (0.93–1.23)a	1.02 (0.87–1.2)a	1.05 (0.90–1.24)a	1.15 (1.0–1.36)a	1.03 (0.87–1.2)a	1.0 (0.84–1.16)a	1.19 (1.0–1.42)a	-
KVS	1.38 (1.16–1.62)b	1.21 (1.10–1.37)b	1.15 (0.99–1.34)a	1.19 (1.10–1.40)b	1.30 (1.11–1.56)b	1.16 (0.99–1.35)a	1.11 (0.99–1.29)a	1.35 (1.14–1.6)b	1.13 (0.98–1.32)a

Table B. **Comparison of field strain probit output at the LT_{99} level.** Resistance ratios (RRs) at LT_{99} and corresponding confidence intervals (in parenthesis) for bed bug strains exposed to 45°C. Significance of RRs was determined using the statistical test described by Robertson et al. [37]. Strains in each column were individually compared to the strains in each row. Within each column RRs that are not connected by the same letter are statistically different. RRs are significantly different if their confidence intervals do not overlap with the number "1". No control mortality occurred during these experiments.

**Strain name**	Harlan	Raleigh	McCall	Hackensack	Richmond	Lafayette	Bradenton	Poultry House	Knoxville
Harlan	-	-	-	-	-	-	-	-	-
Raleigh	0.96 (0.69–1.35)a	-	-	-	-	-	-	-	-
McCall	1.16 (0.93–1.45)a	1.17 (0.86–1.60)a		-	-	-	-	-	-
Hackensack	1.13 (0.80–1.61)a	1.17 (0.86–1.60)a	1.0 (0.72–1.38)a	-	-	-	-	-	-
Richmond	1.13 (0.79–1.62)a	1.17 (0.85–1.61)a	1.0 (0.75–1.0)a	1.0 (0.71–1.40)a	-	-	-	-	-
Lafayette	1.14 (0.81–1.61)a	1.18 (0.87–1.60)a	1.0 (0.73–1.38)a	1.0 (0.73–1.39)a	1.0 (0.72–1.40)a	-	-	-	-
Bradenton	1.19 (0.84–1.68)a	1.24 (0.91–1.68)a	1.0 (0.76–1.45)a	1.0 (0.76–1.45)a	1.05 (0.76–1.47)a	1.0 (0.76–1.43)a	-	-	-
Poultry House	1.16 (0.81–1.64)a	1.24 (0.89–1.72)a	1.0 (0.75–1.49)a	1.06 (0.75–1.49)a	1.06 (0.74–1.51)a	1.0 (0.75–1.47)a	1.0 (0.71–1.41)a	-	-
Knoxville	1.22 (0.86–1.72)a	1.27 (0.93–1.72)a	1.08 (0.78–1.48)a	1.08 (0.78–1.49)a	1.08 (0.78–1.5)a	1.07 (0.78–1.47)a	1.0 (0.75–1.41)a	1.0 (0.73–1.44)a	-
KVS	1.26 (0.90–1.76)a	1.31 (1.0–1.75)a	1.11 (0.82–1.52)a	1.12 (0.82–1.52)a	1.12 (0.81–1.54)a	1.11 (0.82–1.5)a	1.0 (0.78–1.44)a	1.0 (0.76–1.47)a	1.0 (0.76–1.40)a
